# The Role of Transcriptional and Atypical Cyclin-Dependent Protein Kinases in Melanoma

**DOI:** 10.3390/cancers18172726

**Published:** 2026-08-22

**Authors:** Jonatan Kaszubski, Maciej Gagat, Agata Wawrzyniak, Agnieszka Żuryń

**Affiliations:** 1 Vascular Biology Student Research Club, Department of Histology and Embryology, Faculty of Medicine, Collegium Medicum in Bydgoszcz, Nicolaus Copernicus University in Toruń, 85-092 Bydgoszcz, Poland; 324742@stud.umk.pl; 2 Department of Histology and Embryology, Faculty of Medicine, Collegium Medicum in Bydgoszcz, Nicolaus Copernicus University in Toruń, 85-092 Bydgoszcz, Poland; mgagat@cm.umk.pl; 3 Department of Histology and Embryology, Institute of Medical Sciences, College of Medical Sciences of the University of Rzeszów, 35-310 Rzeszów, Poland; awawrzyniak@ur.edu.pl

**Keywords:** transcriptional CDKs, atypical CDKs, melanoma, CDK5, CDK7, CDK8/19, CDK9, CDK11, CDK12/13, CDK16, DNA transcription, cell cycle

## Abstract

Melanoma, a skin cancer with the highest mortality rate, poses a significant medical challenge. Despite the revolution in the treatment of this cancer brought about by the development of immunotherapy and targeted therapies using BRAF/MEK inhibitors, the complex mutation profile and the development of drug resistance compel researchers to seek new solutions. Cyclin-dependent kinases (CDKs), a group of enzymes regulating fundamental processes in every eukaryotic cell, are generating significant interest in the context of potential targeted therapies for melanoma. The best-studied CDKs, responsible for controlling specific phases of the cell-cycle, have been extensively described in the literature, and their inhibition is increasingly used as a treatment for various cancers. However, in addition to the classic cell-cycle CDKs, CDKs regulating transcription can also be distinguished. Other family members responsible for tissue-specific processes are commonly referred to as atypical or untypical CDKs. These include CDK5, which plays a critical role in the nervous system. In recent years, a growing body of research has focused on the role of transcriptional and atypical CDKs in the progression of cancers, including melanoma. However, their precise function remains unclear. This paper will provide an overview of the role of CDKs, other than cell cycle CDKs, in melanoma development and provide a comprehensive understanding of their potential use in future targeted therapies. The advantages and disadvantages of inhibiting these kinases in melanoma therapy will be discussed, as well as the synergies with various molecular pathways analyzed to date.

## Simple Summary

Cyclin-dependent protein kinases (CDKs) regulate various processes in the eukaryotic cell and they constitute therapeutic targets for melanoma treatment. Although the classic cell-cycle-controlling CDKs are highly described in melanoma, the role of the transcriptional and atypical ones still remains unclear. Thus, this comprehensive review is going to present the detailed functions of these kinases, including CDK9 and CDK5, emphasizing their potential role as future targets and synergistic inhibition with other proteins from important signal pathways, including MAPK or PD-1/PD-L1.

## 1. Introduction

Melanoma is considered the most dangerous form of skin cancer. It is characterized by a high mortality rate, and its treatment has always posed a significant medical challenge. It most often develops in skin melanocytes, but rarer forms of melanoma originating in the uvea or mucous membranes have also been described [1]. Cases of melanoma diagnosed in the early stages usually require only surgical treatment. However, if the cancer is diagnosed in later stages, after metastasis, traditional treatment methods are insufficient [2]. 

A true revolution in melanoma treatment was the discovery of its genetic basis and the development of targeted therapies based on characteristic mutations. A particular breakthrough was the introduction of vemurafenib, a serine-threonine kinase B-Raf (BRAF) inhibitor, in 2011 [3]. Unfortunately, the existence of cases with a different mutation profile, as well as the growing problem of drug resistance, compel us to search for new therapeutic methods. In recent years, the role of cyclin-dependent protein kinases (CDKs) in melanoma progression has attracted the attention of many researchers, as well as the possibility of effectively inhibiting cancer cell proliferation using selective inhibitors of the aforementioned kinases [2,4].

CDKs are serine-threonine enzymatic proteins that regulate processes crucial for proper cell proliferation and gene expression via phosphorylation and dephosphorylation. Their activation occurs through the binding of specific proteins, called cyclins, and the attachment of a phosphate group by the CDK-activating kinase (CAK) complex to the T-loop of the C-terminal CDK domain [5,6]. The general structure of CDKs includes N- and C-terminal sequences, which vary in length between proteins, as well as a relatively conserved central kinase domain that performs catalytic functions. The central domain is composed of the following two lobes connected by a hinge region: an N-terminal lobe, primarily in the form of a β-pleated sheet, and a C-terminal lobe, composed of α-helices. This structure allows kinases to effectively bind specific cyclins and attach phosphate groups to other proteins [7]. 

The classification of CDKs based on their functions includes the following three basic groups: “classical” CDKs responsible for cell cycle regulation (CDK1, 2, 3, 4, and 6), CDKs regulating the transcription process (CDK7, 8, 9, 10, 11, 12, 13, and 19), and atypical CDKs (CDK5, 14, 15, 16, 17, 18, and 20), which perform various, mostly tissue-specific processes [5,7,8]. The role of cell-cycle CDKs in melanoma progression, as well as the possibility of their pharmacological inhibition, has been characterized in detail in the literature. However, despite numerous studies conducted in this field, a comprehensive review presenting up-to-date, systematic data on the involvement of the transcriptional and atypical CDKs in the development of this cancer is still lacking. Therefore, this work focuses on filling the gaps in current knowledge about the potential role of “non-classical” CDKs in melanoma and considering their inhibition as a future therapy.

## 2. The Classification of CDKs

### 2.1. Cell-Cycle CDKs

The replication of genetic material and the division into two daughter cells are the processes occurring in all eukaryotic cells, essential for the survival of organisms. The series of biochemical and biophysical reactions occurring in a eukaryotic cell that lead to its division is called the cell cycle [9]. This cycle consists of the M phase, during which the division of the nucleus and cytoplasm occurs, and interphase, which can be divided into G1, S, and G2 phases. During the longest phase of the cycle, G1, the cell increases in size and is prepared for DNA replication, which occurs in S phase. The G2 phase, in turn, verifies whether the duplication of genetic material has occurred correctly and prepares the cell for division. If a cell cannot divide, it enters the G0 resting phase, remaining metabolically active [8,9]. 

To prevent pathological processes, including the development of cancer cells, the cell cycle must be strictly controlled. The best-studied cyclin-dependent kinases, CDK1, 2, 3, 4, and 6, are involved in direct regulation of the cycle, in the form of complexes with cyclins A, B, C, D, and E [10]. If any abnormalities occur, CDK-cyclin complexes are inhibited by specific CDK inhibitors (CDKIs) from the INK4 family (p16INK4a, p15INK4b, p18INK4c, and p19INK4d) and the CIP/KIP family (p21Cip1, p27Kip1, and p57Kip2). These can inhibit the activity of specific CDKs at checkpoints in the cycle, thus blocking cell proliferation. They play a crucial role, as excessive CDK activity results in uncontrolled proliferation and often leads to the development of various types of cancer [6].

**Figure 1:**
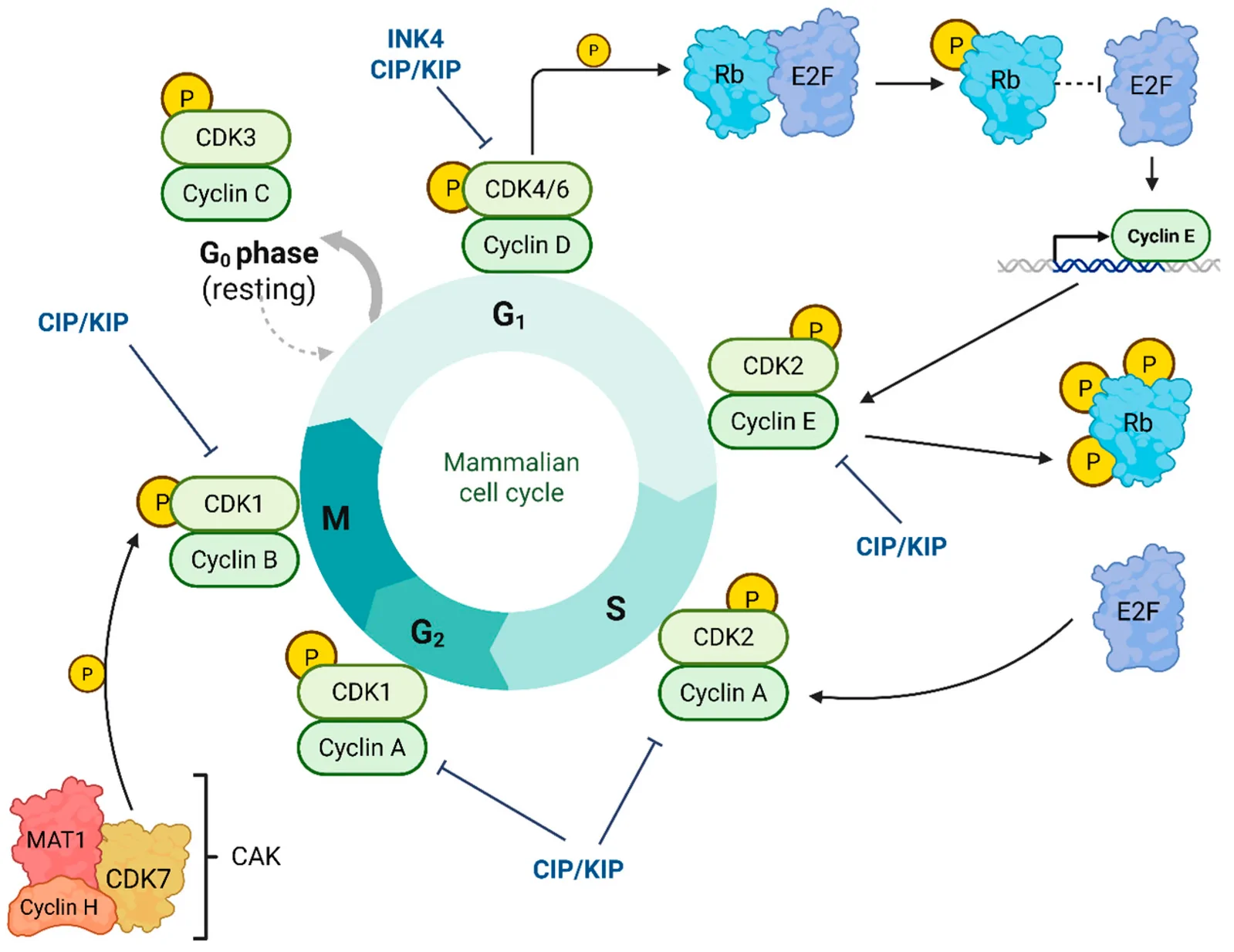
The cell cycle is controlled by specific proteins, including CDKs (CDK1, 2, 3, 4, 6), cyclins (cyclin A, B, C, D, E) and CDKIs from CIP/KIP and INK4 families. The CAK complex, which includes CDK7, cyclin H and MAT1, is responsible for phosphorylating CDK-cyclin complexes in order to activate them. The phosphorylation of Rb by CDK4/6-cyclin D leads to the partial release of E2F and the synthesis of cyclin E. The hyperphosphorylation of Rb results in the full release of E2F and cyclin A production.

The classical course of the cell cycle is illustrated in Figure 1. In the early stages of the G1 phase, CDK4 and CDK6 complexes with cyclin D (specifically, D1, D2, and D3) phosphorylate the retinoblastoma protein (Rb) contained in the Rb-E2F complex. This reaction leads to the release of E2F, which then synthesizes cyclin E. In late G1 and the G1/S transition, the CDK2-cyclin E complex allows the cell to initiate DNA replication. CDK2 then binds to cyclin A, whose synthesis is enabled by further phosphorylation of Rb, enabling progression and completion of the replication process. CDK1 then binds sequentially to cyclins A and B, thus enabling entry into the G2 and M phases, respectively [6,8]. An equally important role is played by the CDK3-cyclin C complex, which allows the cell to progress through the G0 phase by phosphorylation of Rb [5,8]. Indirectly, CDK7 also participates in all the above-described stages of the cycle, which in the CAK complex, together with cyclin H and ménage-à-trois 1 (MAT1), phosphorylates the T loop in cyclin-dependent kinases, enabling their activation [11].

### 2.2. Transcriptional CDKs

Transcription within protein-coding genes, i.e., the synthesis of precursor mRNA (pre-mRNA) on a DNA template, is carried out by the enzyme RNA polymerase II (Pol II), composed of more than a dozen subunits. Transcription can be divided into the following three main stages: initiation, which involves the formation of the preinitiation complex and the initiation of polymerase activity; elongation; and termination. The preinitiation complex is formed out of the transcription factors, large protein complexes that enable Pol II to bind to the template as follows: TFIIA, TFIIB, TFIID, TFIIE, TFIIF, and TFIIH. Together with Pol II, they form the basic transcription apparatus [6,12]. 

**Figure 2:**
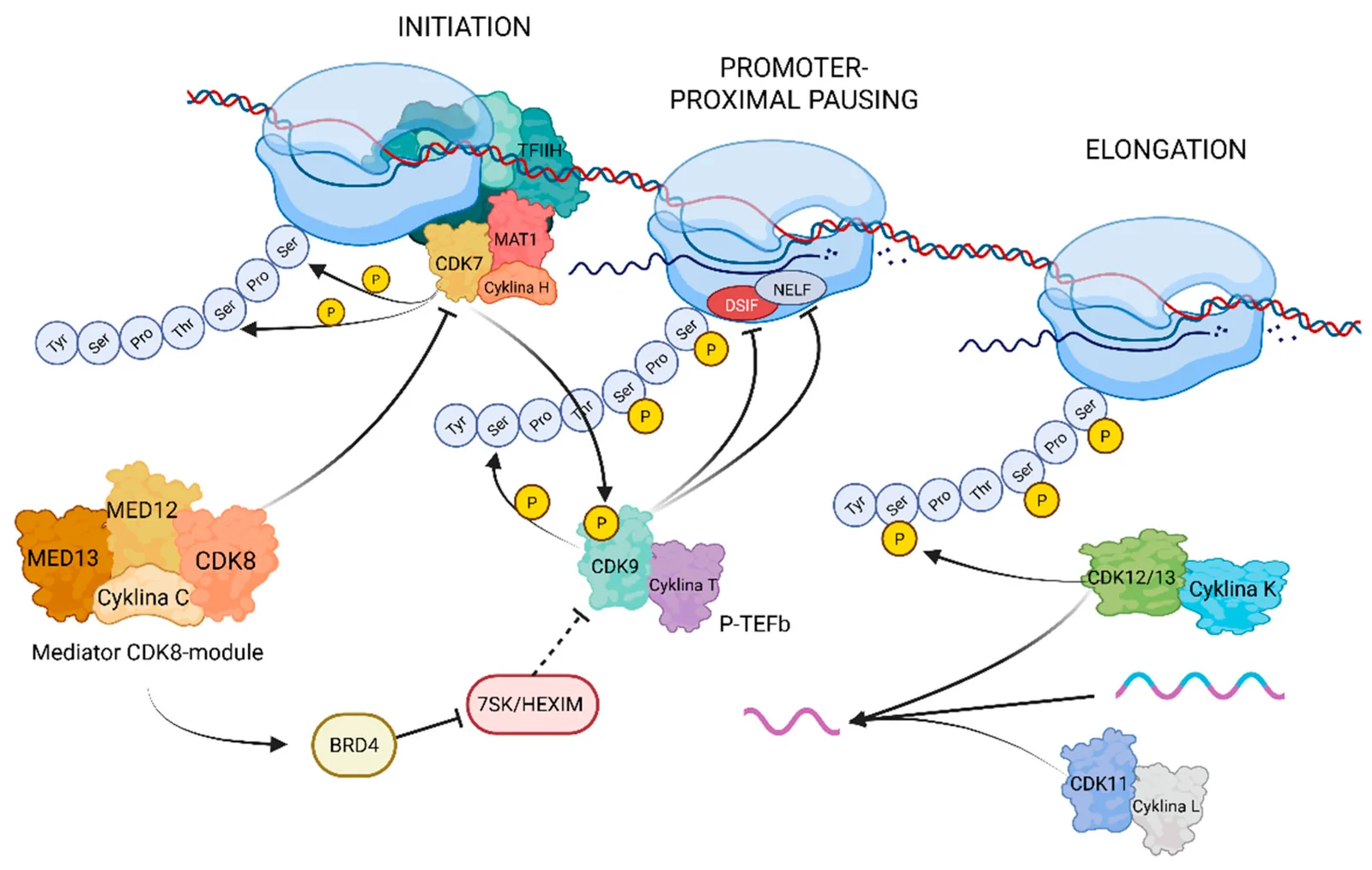
Transcription process is regulated by large complexes called transcription factors, which are composed of various proteins, including the transcriptional CDKs (CDK7, 8, 9, 11, 12, 13, 19), which phosphorylate the amino acid sites on CTD of Pol II.

Although CDKs involved in cell cycle regulation are the best studied, there is also a large group of transcription CDKs (Figure 2). CDKs 7, 8, 9, 10, 11, 12, 13, and 19, as the components of transcription factors, are responsible for attaching phosphate groups to amino acid residues in the C-terminal domain (CTD) of RNA polymerase II (Pol II). The CTD consists of repeating fragments with the sequence Tyr-Ser-Pro-Thr-Ser-Pro-Ser, of which serine residues are the most important in the context of CDK phosphorylation [5,6,10]. A list of transcription CDKs and their key features is presented in Table 1.

CDK7 is a very important protein kinase, which is responsible for controlling both transcription and cell cycle. Along with its binding partner cyclin H and ménage-à-trois 1 (MAT1), it constitutes the CDK-activating kinase (CAK). This complex, as a part of the transcription factor IIH (TFIIH), plays a significant role in the transcription process, as well as in the proper cell cycle progression, as CDK7 phosphorylates the T-loop of the other CDKs, therefore activating them [14]. CDK7 plays two essential roles in transcription. The first one is the phosphorylation of serine 5 (less often serine 7) on the CTD, which allows the polymerase to dissociate from the preinitiation complex [12,15,16]. The second function of CDK7 is revealed when the polymerase pauses 50–70 nucleotides downstream of the transcription start site. This situation, called promoter-proximal pausing, is released by the positive transcription elongation factor (P-TEFb), which comprises CDK9 and cyclin T. P-TEFb is activated by phosphorylation of the T-loop of CDK9, initiated by CDK7, within the CAK complex [12,17]. CDK9 is a transcriptional protein kinase, which is able to bind with cyclin T1, T2a, T2b or cyclin K. It occurs in the cell in the following two isoforms: CDK9^42^ and, longer of 117 additional amino acids, CDK9^55^ [18,19]. 

During the pause, P-TEFb activity is suppressed by the U1 small nuclear ribonucleoprotein (snRNP) 70 and the HEXIM1/2 protein. Therefore, P-TEFb activation is only possible when the 7SK/HEXIM inhibitory complex is inactivated by bromodomain-containing protein 4 (BRD4), following histone acetylation. P-TEFb leads to the transition from transcription initiation to elongation via three main reactions. The first is the phosphorylation of the CTD at serine 2, while the other two involve the inactivation of factors that inhibit transcription initiation. DRB sensitivity-inducing factor (DSIF) is converted into a positive elongation factor, while negative elongation factor (NELF) is dissociated from the polymerase by P-TEFb [12,20]. 

Transcription is subject to both positive and negative control by the high-molecular-weight Mediator protein complex, which consists of 25–30 subunits grouped into the following four main modules: head, middle, tail, and the CDK8 kinase module. Each of these modules can perform distinct functions. The CDK8 kinase module is a domain of Mediator characterized by enzymatic catalysis and includes CDK8, cyclin C, MED12, and MED13 [21]. The best-studied role of Mediator is to form specific “bridges” between transcription factors bound to their regulatory sites and elements of the basal transcription apparatus, thus enabling regulation of expression. CDK8 or its paralog CDK19 can negatively regulate transcription by phosphorylating serine residues 2 and 5 in the CTD of Pol II, thereby preventing the polymerase from binding to the preinitiation complex [22,23]. CDK8/19 are also responsible for phosphorylation of the CDK7-cyclin H complex, which inhibits transcription initiation [24,25]. However, CDK8 should not be considered exclusively a transcriptional repressor [26]. CDK8-/19-containing Mediator complexes participate in enhancer–promoter communication and chromatin organization [27,28]. What is more, in some conditions, such as hypoxia, CDK8 may act as a positive regulator of transcription by promoting the activity of BRD4 and P-TEFb [29]. Moreover, CDK8 and CDK19 may exert distinct functions in transcriptional regulation, with CDK8 acting through its kinase activity and CDK19 also contributing through kinase-independent scaffolding functions. There were also reports that CDK8/19 may regulate the transcription memory in response to heat stress [30].

CDK8 and CDK19 show substantial functional redundancy due to their high sequence similarity. They also both function within the Mediator kinase module. However, they are not completely functionally interchangeable, as due to the small differences in the C-terminal domain, they may sometimes play distinct functions during transcription process, with CDK8 acting through its kinase activity and CDK19 also contributing through kinase-independent scaffolding functions [31]. This fact poses a challenge for pharmacological targeting, as the high similarity of their catalytic domains makes selective inhibition of either paralog difficult. Consequently, most currently characterized CDK8/19 inhibitors, including cortistatin A, CCT251545 and SEL120, inhibit both kinases with comparable potency rather than selectively targeting one paralog [32,33,34].

CDK12 and CDK13 are responsible for maintaining the appropriate elongation rate. Despite significant differences between them, both bind cyclin K and phosphorylate serine 2 in the CTD chain [13,35,36]. In this way, they increase both the number of nucleotides added during a single binding of the polymerase to the template (processivity) and the number of nucleotides added per second (speed). CDK12/13 also prevent premature termination and the formation of incomplete transcripts by blocking alternative intronic polyadenylation sites. Moreover, it is also involved in the phosphorylation of cyclin E1, therefore leading to the attenuation of its binding to CDK2, which makes CDK12 a DNA replication regulator [35,37,38]. CDK12 also regulates expression of the genes, responsible for the pre-replication complex formation. Furthermore, CDK12 and CDK13 also participate in the regulation of intron excision from pre-mRNA (splicing) and translation [35,37]. 

The splicing process is also regulated by the CDK11-cyclin L complex, which also participates in the control of the expression of genes encoding histone proteins and apoptosis. CDK11 also interacts with the TFIIF and TFIIS factors [8]. CDK11 exists in over 20 different isoforms, which appear to be an effect of the caspase cleavage or the alternative splicing of the N-terminal coding exons. The three main isoforms, CDK11p110, CDK11p58 and CDK11p46, are able to bind with cyclin L, therefore creating its active form. Similarly to CDK9, this kinase phosphorylates CTD of Pol II and promotes the transcription elongation. However, the deeper research on the biological function of this protein brought us the insight into the fact that CDK11 regulates much more significant various processes, like the proper chromosome segregation or pre-mRNA splicing. Therefore, it is also involved in many pathological conditions, including the tau protein phosphorylation in Alzheimer’s disease or replication of HIV-1 virus [39,40,41]. CDK10 and cyclin M, in turn, are responsible for the negative regulation of ETS2 proto-oncogene-dependent transcription through phosphorylation and degradation of the ETS2 N-terminus. The precise role of CDK10 is unknown, although there are reports in the literature of its involvement in cilia formation and cytoskeletal organization [12,20]. The information on pre-clinical studies relevant to the transcriptional CDKs in melanoma are concluded in Table 2.

### 2.3. Atypical CDKs

Some enzymes belonging to the cyclin-dependent kinase group differ significantly from classical CDKs in both structure and function. Instead of directly controlling the cell cycle or transcription, they regulate various, often tissue-specific, processes. Such kinases are referred to as untypical or atypical CDKs. Despite the lack of an official classification in the current literature, this group most often includes CDK5, CDK14–18, and CDK20 [5,10]. The classification based on the sequence of the cyclin-binding region, the most important features, and the involvement of atypical CDKs in pathological conditions are presented in Table 3.

CDK5 is the most studied atypical cyclin-dependent kinase. Of all the kinases in this group, it is most distinctive in that it does not require T-loop phosphorylation for activation and does not bind any cyclins. Instead of cyclins, CDK5 binds primarily p35 and p39 proteins. However, the most recent studies revealed that this can also occur in a functional complex with cyclin B1 to regulate mitosis [57]. It is also insensitive to CDKIs from the CIP/KIP family [53]. CDK5 primarily demonstrates activity in the nervous system. Through various pathways, it stimulates neuronal migration and participates in neurogenesis. It plays a key role in axonal growth and the formation of the neuronal cytoskeleton. CDK5 also controls Ca^2+^ ion influx, thus modulating the synaptic plasticity, which significantly influences the memory [58]. Furthermore, CDK5 influences cell adhesion and apoptosis, as well as indirect cell cycle arrest through its interaction with transforming growth factor beta (TGF-β), Rb protein, or E2F [59]. In pathological conditions, CDK5 may drive the development of various types of cancers, as well as Alzheimer’s and Huntington’s diseases [60]. 

Other atypical CDKs are often classified in the literature into the following two groups: PFTAIRE (CDK14, 15) and PCTAIRE (CDK16, 17, 18), based on the amino acid sequence of the cyclin-binding region. Some of them are very poorly studied. Their primary binding partner, particularly in the case of CDK14 and CDK16, is cyclin Y [54]. CDK16 is the only protein in the PCTAIRE group encoded on the X chromosome. Although it is not a classic cyclin-dependent kinase that directly regulates the cell cycle, it indirectly influences many stages of the cell cycle by stimulating the JAK/STAT or Akt/mTOR pathways [55]. The remainder of this review will discuss evidence for the involvement of CDK16 in melanoma development. It is currently known that CDK16 is synthesized primarily in the brain, testes, and muscles, thereby regulating various processes such as spermatogenesis, vesicle transport, autophagy, actin filament organization, apoptosis, glucose transport and myocyte maturation. According to some studies, CDK16 also participates in apoptosis and indirect cell cycle control, thus contributing to the development of cancers such as lung, breast, and prostate [54,55]. 

Atypical CDKs also include a kinase with an amino acid sequence similar to CDK7, namely CDK20. The classification of CDK20 as a transcriptional kinase, due to its similarity to CDK7, is controversial, but it is known that it exhibits CAK activity towards CDK2 and is expressed in various cancers [56]. In the following chapters of this work, the role of these proteins in the development of melanoma and their potential in the treatment of this disease will be presented. The information on pre-clinical studies relevant to the atypical CDKs in melanoma are concluded in Table 4.

## 3. The Genetic Landscape of Melanoma

**Figure 3:**
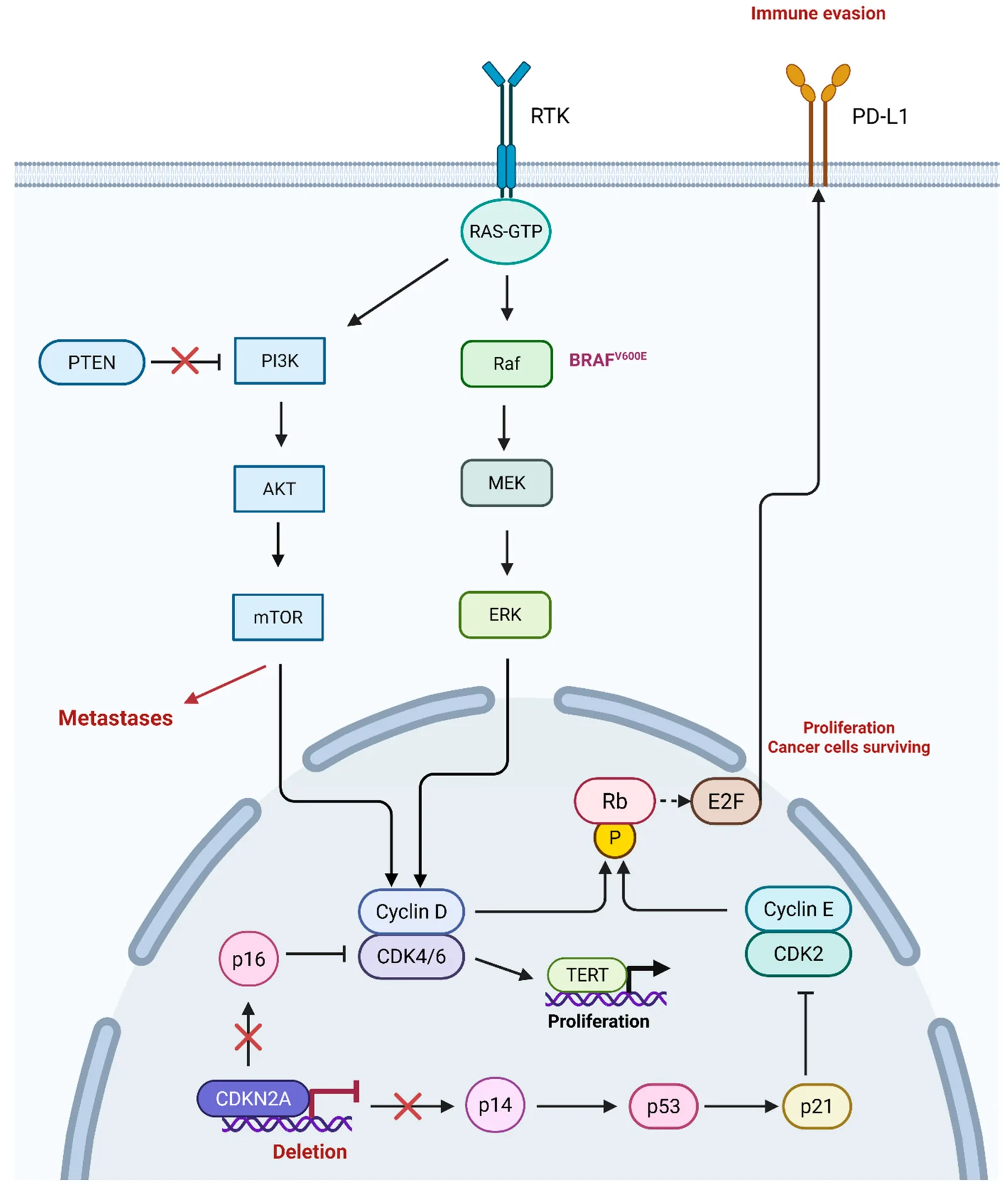
Various molecular pathways, including PI3K/Akt/mTOR and MAPK, are involved in the progression of melanoma. Most cases are driven by somatic mutations, like BRAFV600E in the BRAF gene or CDKN2A deletion in the p16INK4a-cyclin D-CDK4/6-Rb pathway.

Melanoma develops in melanocytes, epidermal cells that synthesize the pigment melanin from tyrosine, which is then transported to keratinocytes to absorb ultraviolet (UV) radiation and thus protect the skin. Excessive exposure to UV radiation often leads to the gradual accumulation of mutations in the genome, which can result in cancer development. This is the case in approximately 80% of melanoma cases; the remaining cases are mostly genetic [66,67]. Mutations in melanoma are mostly somatic mutations, meaning they are acquired during lifetime and do not pass on to an offspring [68]. Approximately 50% of melanoma cases are associated with mutations in the proto-oncogene encoding BRAF kinase, a member of the RAF protein family. In most cases, it is the BRAFV600E point mutation, which leads to hyperactivation of the mitogen-activated protein kinase (MAPK) pathway [69]. This pathway, also known as RAS/RAF/MEK/ERK, is responsible for transferring signals from the cell surface to the nucleus [66,68]. The mutation results in continuous activity of the pathway without external signals, leading to excessive, uncontrolled cell proliferation (Figure 3).

Another important member of the RAS/RAF/MEK/ERK pathway is the NRAS protein, a member of the small GTPases (RAS) family. In its active, guanosine triphosphate (GTP)-bound form, it phosphorylates and recruits RAF proteins. Constitutive activation of the NRAS protein, resulting from mutations, is a very common defect in melanoma, following BRAF mutations [68]. In cutaneous melanoma, activating mutations in BRAF or NRAS are common and are generally mutually exclusive, whereas a substantial subset of tumors lacks alterations in either gene [70,71,72]. In contrast, BRAF and NRAS mutations are considerably less frequent in acral, mucosal melanomas and hardly ever observed in uveal melanoma [70,73]. Genetic or epigenetic inactivation of the phosphatase and tensin homolog (PTEN) tumor suppressor gene appears to be equally dangerous, leading to constitutive activation of the PI3K/Akt/mTOR pathway and the occurrence of metastases [67].

Cell-cycle disorders caused by mutations in genes encoding CDKs, cyclins, or their inhibitors very often accompany the development of melanoma. The p16INK4a-cyclin D-CDK4/6-Rb pathway is frequently dysregulated in melanoma and plays an important role in the regulation of cell-cycle progression [74,75]. In a genomic analysis of primary melanomas and adjacent precursor lesions, biallelic inactivation of CDKN2A was observed exclusively in invasive melanomas, suggesting an association with progression to an invasive phenotype [76]. The vast majority of these are deletions of the CDKN2A gene, which encodes two proteins. One of them is p16INK4a, an inhibitor that inhibits phosphorylation of Rb protein by the CDK4/6-cyclin D complex [4]. The second protein encoded by CDKN2A is p14ARF, which binds and inactivates the HDM2 protein, thereby stabilizing the p53 protein, an important tumor suppressor [68]. In pathological conditions, p53 protein maintains high levels of p21Cip1, which in turn inhibits the CDK2-cyclin E complex. CDKN2A deletion therefore results in continuous phosphorylation of the Rb protein and a lack of control at the G1/S point of the cell cycle [4,68]. In many cases, cell-cycle control disorders are caused by amplification of the gene encoding cyclin D, CCND1, caused by mutations in BRAF and phosphoinositide 3′ kinase (PI3K) [2,4]. Cell-cycle disorders in melanoma are often accompanied by mutations in programmed death ligand 1 (PD-L1) and TERT, the gene encoding the catalytic subunit of the enzyme responsible for telomere elongation, called telomerase. Correlations of CDK4 and TERT mutations are particularly noticeable in mucosal melanoma [4,68,77].

Although melanoma was once considered a highly fatal disease, subsequent discoveries in immunotherapy and targeted therapies have revolutionized the treatment of this cancer. While the stage 1 melanomas are often treated with surgery, chemotherapy, or radiotherapy as the main treatment options, antibodies against the PD-1 antigen and inhibitors targeting BRAF and mitogen-activated protein kinase (MEK) mutations, such as vemurafenib, dabrafenib, and trametinib, have proven particularly effective for advanced melanoma [78,79]. Pharmacological drugs targeted at BRAF are frequently used in combination with MEK inhibitors [80,81]. However, even these have not fully solved the problem of patients with melanoma without the aforementioned mutations, including acral or mucosal melanoma, because BRAF mutations are much more frequent in cutaneous melanoma [70,82,83]. Increasing resistance to current drugs has also continued to pose a challenge. Numerous studies on specific CDKIs, particularly those targeting CDK4/6 and CDK2, have proven their potential. Although their use in monotherapy does not seem promising, due to relatively low efficiency and the existence of cytotoxicity, their use in synergy with BRAF/MEK inhibitors or immunotherapy is attracting attention [84]. Currently ongoing and already completed preclinical and clinical trials of the first and second phases focus mainly on the evaluation of the activity of CDKIs, such as those used in the treatment of breast cancer: palbociclib, abemaciclib, or ribociclib, which are targeted at cell cycle-controlling CDKs [84,85,86]. The challenge remains to find new synergies of CDKIs with other drugs in order to minimize toxicity and achieve the best possible therapeutic effects [85]. However, compared to studies on the role of CDK4/6 in melanoma and the potential of therapies targeting these kinases, much less frequently reported studies on transcriptional or atypical CDKs. The subsequent chapters of this work will therefore focus on the involvement of CDKs other than the classic, cell-cycle-regulating CDKs in melanoma, as well as their potential as future targets for new therapies.

## 4. The Role of Transcriptional CDKs in Melanoma

### 4.1. The Role of CDK7 in Melanoma

The early research did not show any sign of CDK7 overexpression in melanoma cells. Several studies reported that in both normal and melanoma cells, CDK7 and its binding partner cyclin H were located mainly in the nucleus, and their expression was nearly the same in both types of cells [87]. According to the later analysis, the cyclin H level was slightly decreased after the melanoma cell proliferation was suppressed by the lack of adhesion to substratum. Despite this fact, there were still no changes in CDK7 expression [88]. No decline of this kinase was observed after the betulinic acid-mediated inhibition of the melanoma cells either [89].

However, despite the initial lack of any important observations, some later studies proved the involvement of CDK7 in melanoma. CDK7 correlated with the microphthalmia-associated transcription factor (MITF), an oncogenic transcription factor, which plays a significant role in the melanoblast proliferation and melanin production [42]. The research points out the significant correlation between MITF and CDK7, describing them as novel targets for melanoma treatment. CDK7 associates with super-enhancer regions regulating the MITF and SOX10 genes. These regulatory elements support the high transcriptional activity of genes that are essential for the melanocytic phenotype and melanoma cell survival. According to another study, which focused on DNA transcription and repair in human melanocytes, MITF controls CDK7 expression doubly [90]. Besides the direct regulation, MITF can induce the transactivation of far upstream binding protein 2 (FUBP2), which in turn is involved in the pulse regulation of another transcription factor c-MYC, a MITF structural homolog. Therefore, CDK7 expression in melanocytes is dependent on MITF and c-MYC encoding genes, which are both proto-oncogenes. This fact highly confirms the role of CDK7 in melanoma progression.

The suppression of CDK7, with the use of THZ1, a selective CDK7 inhibitor, inhibited phosphorylation of both Pol II CTD and the T-loop of CDK9. Therefore, melanoma tumor growth was arrested by THZ1 in vitro and in vivo. The inhibition of CDK7 also led to the downregulation of the oncogenic transcription factors MITF and SOX10 encoding genes, by disrupting their associated super-enhancers, large clusters of enhancers driving high expression of oncogenic genes [42].

From another perspective, CDK7 promotes the expression of MITF, which in turn represses GATA-binding factor 6 transcription factor (GATA6), by binding its intronic regulatory sequence [44]. GATA6 controls the transcription program that includes genes responsible for melanoma survival and tolerance for drugs, including BRAF/MEK inhibitors, therefore inhibiting CDK7 that mediately leads to drug resistance. The CDK7 inhibition was discovered to promote the de-differentiation of melanocytic-type melanoma cells into mesenchymal-like cells, which frequently leads to drug tolerance and metastases. This effect shows that targeting CDK7 in monotherapies may be dangerous. In future studies, these discoveries should be used to find a synergistic therapy, as blocking only CDK7 may bring the opposite to predicted effects. 

SNS-032, a CDKI with activity against CDK2, CDK7 and CDK9, was tested on the uveal melanoma cells and xenografts [43]. Uveal melanoma is an extremely rare disease, with a high liver metastatic risk, which frequently remains a lead cause of the patients’ death [91]. Consulting this fact, the researchers analyzed the effect of CDKI not only on tumor growth, but also on cell invasiveness, motility and metastases, which were all found to be suppressed by the SNS-032 [43]. Apoptosis induction and arrest of cell proliferation were correlated with the abrogated transcription of YAP, a transcriptional cofactor involved in tumorigenesis. In the xenografts, CDKI inhibited the stemness-related protein Krüppel-like factor 4 (KLF4), which led to the elimination of the stem-like cells, whereas the cell invasiveness was arrested through the matrix metalloproteinase 9 (MMP9). The liver metastases were observed to be dependent on the actin polymerisation and the activity of RhoA GTPase, which is known to play a crucial role in the cytoskeletal organization. Transcription of a gene that encodes this GTPase, RhoA gene, was inhibited by SNS-032 through the suppression of c-MYC. To sum up, the analysis revealed that the expression levels of both CDK7 and CDK9 are increased in uveal melanoma and that inhibition of these kinases arrest the cancer cell proliferation and invasiveness through various mechanisms. These findings highlight the potential of transcriptional CDKs as therapeutic targets in uveal melanoma and suggest that their inhibition may affect both tumor growth and metastatic progression.

**Figure 4:**
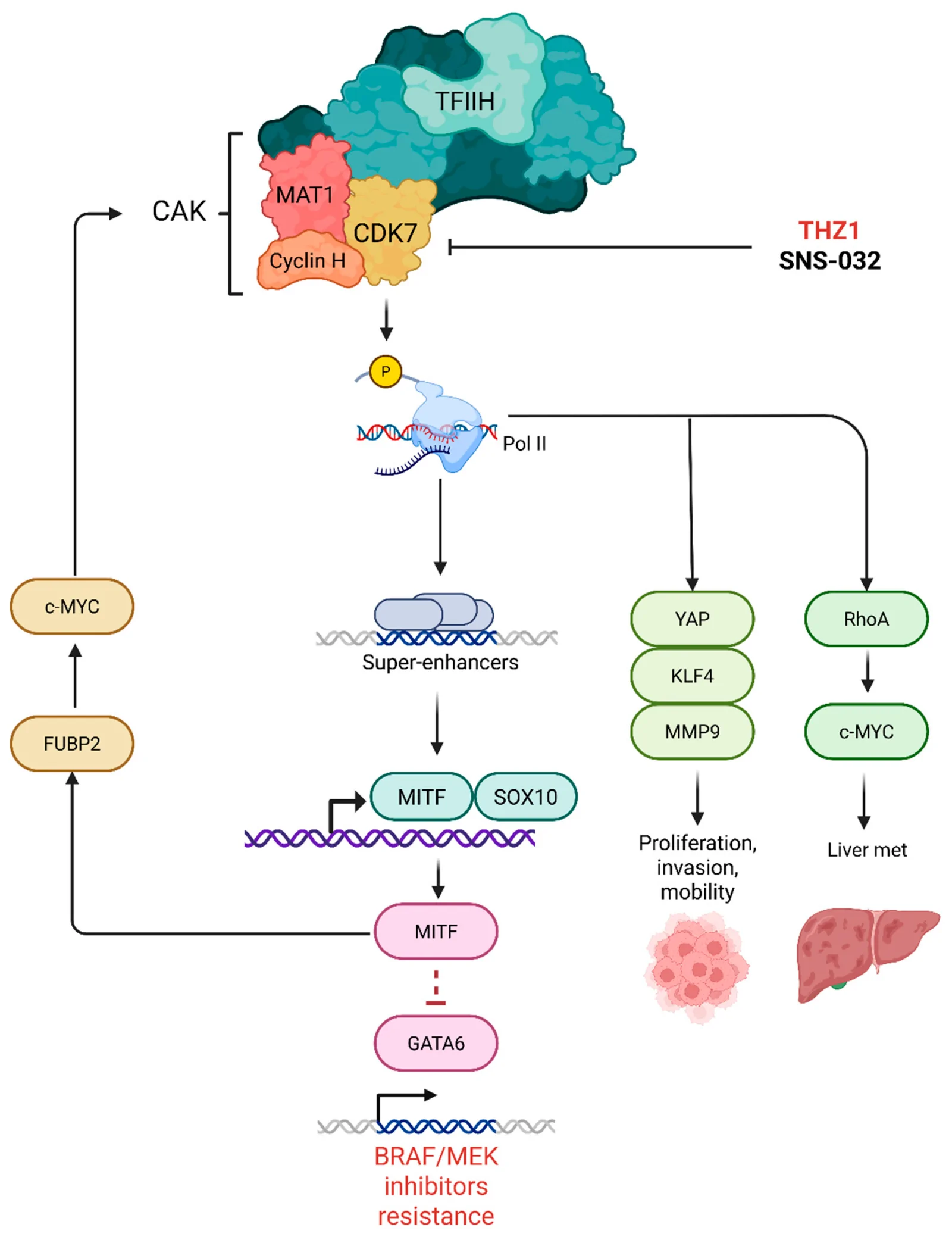
CDK7, along with cyclin H and MAT1, forms a CAK complex, which is a part of TFIIH. In melanoma, CDK7 is responsible for cell proliferation, mobility and liver metastases. It can also regulate the MITF and SOX10 transcription factors through the super-enhancers of those genes. CDK7 can be inhibited by SNS-032 and THZ1 inhibitors. However, the use of THZ1 in monotherapy may result in the BRAF/MEK inhibitors resistance.

Taken together, current evidence suggests that CDK7 contributes to melanoma biology primarily through the regulation of transcriptional programs rather than through simple changes in its expression level. Its functions include supporting Pol II-dependent transcription, maintaining MITF-/SOX10-associated regulatory programs, interacting with MITF-dependent transcriptional networks, and influencing melanoma-cell proliferation, phenotype, invasiveness, and metastatic potential. These findings provide a biological rationale for targeting CDK7. However, the emergence of adaptive transcriptional programs following its inhibition indicates that CDK7-directed treatment may be more effective when combined with other therapeutic approaches. The findings relevant to the role of CDK7 in melanoma are presented in Figure 4.

### 4.2. The Role of CDK8/19 in Melanoma

To our knowledge, the first mention of the involvement of CDK8 in the progression of melanoma appears in a study by Kapoor et al. [92,93,94,95]. Knockdown of the histone variant macroH2A (mH2A) was found to promote proliferation and migration of melanoma cells, as well as tumor growth and metastases, through the upregulation of CDK8. Analogically, the loss of CDK8 arrested the melanoma cell proliferation in G2/M cell cycle phase. CDK8 was suggested to give this effect within the Mediator complex, as the knockdown of MED12 brought similar results. Furthermore, the researchers analyzed the CDK8 and mH2A levels in the melanoma samples, taken from patients, to find an inverse correlation between those two elements. Similar results were described when the overexpression vectors of mH2A were transferred into melanoma cells and then analyzed by quantitative polymerase chain reaction (PCR) [96]. The expression of CDK8, along with CDK4/6 and cyclin D, was found to be inhibited by the overexpression of mH2A, which correlated with G2/M arrest and apoptosis. All these facts indicate that CDK8 and mH2A have an antagonistic relation and are both involved in melanoma progression.

CDK8 was also reported to mediate the phosphorylation of the transcription factor STAT1 at serine727 in natural killer (NK) cells [97]. NK cells are immune cells, which are responsible for the immune defense against transformed cells. The mutation of this phosphorylation site, Stat1-S727A, was observed to enhance cytotoxicity of NK cells. Stat1-S727A mice appeared to have enhanced resistance to melanoma tumor formation in comparison to wild-type mice. The mice with the mutation were also characterized with an increased overall survival, which suggests CDK8 as a potential target in melanoma therapy, with the use of the NK cell stimulation. The NK cell cytotoxicity was also confirmed to be increased by the CDK8 deletion, through the enhanced perforin level [98]. The analysis revealed that the lung nodules, formed after the injection of melanoma cells, were suppressed more effectively in the mice with lower CDK8 expression level in NK cells. Another research included targeting the NK cells with a selective CDK8/19 inhibitor, BI-1347, in order to inhibit the STAT1-S727 phosphorylation [99]. The mice bearing melanoma xenografts had a significantly enhanced response rate and survival, as well as the lower tumor burden after the BI-1347 treatment.

The expression of CDK8 was reported to be heterogenous and associated with short survival [100]. CDK8 encoding gene, next to the CDKN2D, was also identified as a target of the preferentially expressed antigen of melanoma (PRAME) gene in the embryonic stem cells (ESCs) [101]. Moreover, the in silico target prediction analysis revealed CDK8 as one of the predicted targets for the most active compounds, which were selected for the treatment of melanoma and non-melanoma skin cancers [102]. However, these findings have not been scientifically proven yet. To sum up, the role of CDK8 in melanoma progression is mostly predictable to be correlated with the transcription factor STAT1 in NK cells and with mH2A histone variant. Future pre-clinical trials should highlight the involvement of this kinase in the pathological processes and its potential as a target.

### 4.3. The Role of CDK9 in Melanoma

The recent studies indicate that CDK9 may be a very promising target in melanoma therapy. Melanoma cells were reported to be inhibited by dinaciclib – a molecule targeted not only at CDK1, CDK2 and CDK5, but also at CDK9 [103]. There are also reports that CCT068127, novel CDKI targeted at CDK9 and CDK2, reduces the melanoma cell proliferation with higher antiproliferative activity by 50%, in comparison to roscovitine, which is also targeted at CDK1, 5 and 7 [104]. Several other studies have also proposed CDK9 inhibition for treating melanoma; for example, Atuveciclib, a P-TEFb inhibitor with an anti-leukemia potential, was identified with an antiproliferative effect against melanoma [105]. Moreover, the suppression of uveal melanoma cell proliferation and liver metastases by SNS-032 was related with the inhibition not only of CDK7, but also of CDK9 [43]. However, another study revealed that the chronic inhibition of CDK9 may constitute the reason behind the resistance for immunotherapy [106].

The inhibition of CDK9 was discovered to have a synergistic effect with PLX4032, a BRAFV600E inhibitor [45]. After PLX4032 was targeted at the melanoma cells with BRAFV600E mutation, only parts of the cells were observed to be dead. The surviving cells (aka. SUR), which appeared to be very similar to senescent cancer stem cells, were treated with histone deacetylase (HDAC) inhibitors and CDK9 inhibitor, CDKI-73, in order to find if the senescence and drug resistance of the SUR cells result from epigenetic regulation. Both SUR and parental melanoma cells were observed to be inhibited by CDKI-73, which was also discovered to induce apoptosis in both of those cell types. Moreover, the CDK9 inhibitor had a synergistic effect with PLX4032 and HDAC inhibitors in a 3D melanoma growth model. This discovery suggests the future use of CDK9 inhibitors in correlation with BRAF inhibitors. This correlation can be a solution to the resistance for BRAF/MEK inhibitors and therefore constitutes an important synergy in future clinical trials.

A significant relation between the inhibition of CDK9 and p53 tumor suppressor activation was revealed [46]. MDM4 oncogene level was decreased, therefore activating p53, after A375 melanoma cells were treated with the following several CDKIs: dinaciclib, flavopiridol, roscovitine, AT-7519, SNS-032, and DRB, all targeted at CDK9. What is interesting is that those CDKIs did not affect the mouse double minute 2 homolog (MDM2) level as much as MDM4, although MDM2 had always been considered the key negative p53 regulator. Moreover, the research presents that the small-molecule p53-MDM2 interaction inhibitor, nutlin-3a, works synergistically with atuveciclib, a selective CDK9 inhibitor. Those two molecules were found to decrease the MDM4 level and enhance the p53 activation, therefore leading to the cell death. Štětková et al. also discovered that CDK9 is responsible for controlling the MDM4 level in human embryonic stem cells (hESCs), where p53 expression is always precisely controlled in order to prevent differentiation [46]. The researchers suggest CDK9 and MDM4 are somehow responsible for controlling the maintenance of genomic stability in hESCs, as well as DNA repair in pluripotent cells; however, more research needs to be conducted in order to confirm this hypothesis.

Selective CDK9 targeting was found to be an effective solution for inhibiting the growth of melanomas lacking in BRAF/NRAS/NF1 mutations [47]. As BRAF/NRAS wild-type (WT) cutaneous and uveal melanoma cannot be treated with BRAF inhibitors, the new methods for their treatment remain a search target [107]. It was discovered that inhibition of CDK9 is more effective in suppressing BRAF/NRAS WT uveal and cutaneous melanoma than the types burdened with BRAF/MEK mutations [47]. The same results were observed after targeting CDK9 with its degrader TS-032 and a selective inhibitor NVP-2. RNA sequencing of cells targeted with NVP-2 revealed that the biggest expression changes included the genes regulated by proliferation-related transcriptional factors, especially by E2F family members. The E2F family is a group of proteins that are directly involved in the G1/S transition in the cell cycle, as well as the processes of apoptosis and DNA replication [108]. According to the information from the Cancer Genome Atlas human melanoma tumor data, the researchers came to the conclusion that two proteins from the family mentioned above, E2F1 and E2F2, are potentially involved in BRAF/NRAS/NF1 WT cutaneous and uveal melanoma progression [47]. As CDK9 promotes transcription elongation, its inhibition leads to cell cycle exhaustion of E2F family members, eventually providing the suppression of proliferation and cell death. 

A selective CDK9 inhibitor, CDKI73, arrested the growth of melanoma cells synergically with a BET inhibitor, independent of their mutation status of BRAF and NRAS [48]. These two combined inhibitors induced caspase-dependent apoptosis in the cancer cells, which was associated with mitochondrial membrane depolarisation. Characteristically to that type of apoptosis, a cleavage of Poly [ADP-ribose] polymerase 1 (PARP1), an enzyme involved in DNA repair, was observed. Furthermore, CDKI73 combined with a BET inhibitor led to the downregulation of anti-apoptotic proteins, including BCL2, BCLXL and MCL1. Among the downregulated genes, there was also an oncogene c-MYC and E2F encoding gene. Moreover, the research also included a gene set enrichment analysis (GSEA) on RNA-sequencing from cell lines treated with the inhibitors mentioned above. The analysis revealed the decrease in proteins, which regulate the G2/M checkpoint in the cell cycle. Overall, the study presents the synergistic role of CDK9 and BET in the processes of apoptosis and cell cycle, as well as the potential therapeutic option of inhibiting those two proteins. 

The inhibition of CDK9 was also proved to promote apoptosis and cell cycle arrest, when Yin et al. targeted a selective CDK9 inhibitor LS-007 at B16F10 melanoma cells [49]. The ratio of a pro-apoptotic protein Bax to an anti-apoptotic Bcl-2 was significantly increased, as well as the expression of an active form of caspase-3 and PARP, while the Mcl-1 level was decreased. However, besides apoptosis promotion, LS-007 also decreased a tumor volume and weight in vivo and led to the downregulation of a proliferation marker Ki-67. Furthermore, the inhibition of CDK9 was discovered to promote the polarization of macrophages into an antitumor phenotype in the tumor microenvironment. All these findings indicate multiple roles of CDK9 in melanoma progression, as its inhibition not only promotes apoptosis and suppression of cell cycle but also changes tumor microenvironment.

**Figure 5:**
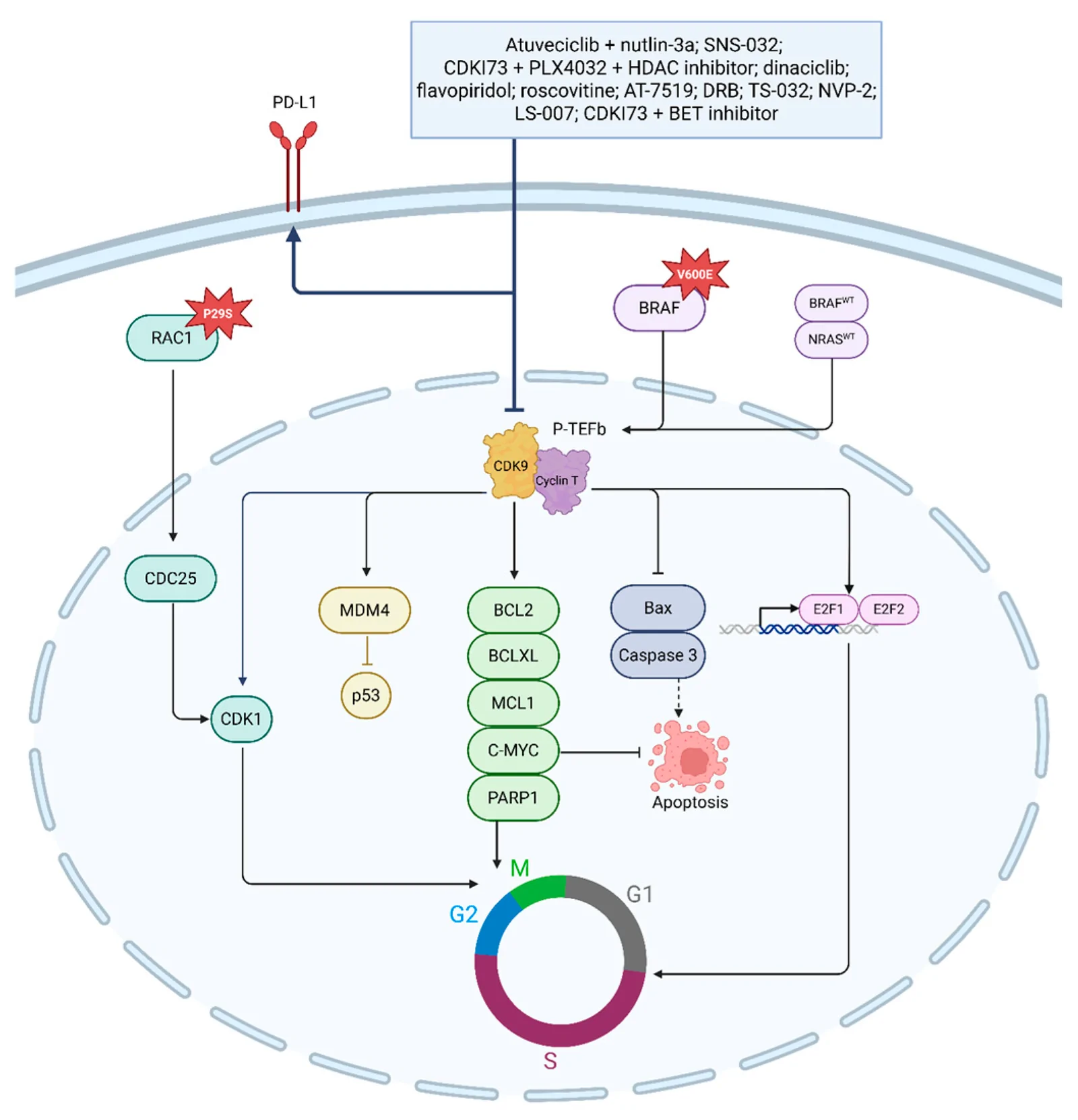
P-TEFb (CDK9 and cyclin T) regulates the progression of melanoma through various pathways, including activating the anti-apoptotic proteins like BCL2 and inhibiting the pro-apoptotic proteins, like Bax and caspase 3.

CDK9 was identified as a novel target in RAC1-driven melanoma [50]. RAC1^P29S^ is a common mutation, which may constitute a cause of chemotherapy resistance or insensitivity to inhibitors. The researchers conducted RNA-sequencing of RAC1^P29S^ melanoma cell line, in order to find the biological mechanism behind this mutation. RNA-sequencing analysis was combined with a multiplexed inhibitor bead-mass spectrometry to investigate the relations between the genomic level and the protein profile. The melanotic expression of RAC1^P29S^ was found to promote G2/M transition and CDK1 phosphorylation of the melanoma cells, through the CDK9 activation, which was proved by the CDK9 inhibition. Furthermore, the combination of a CDK9 inhibitor and an anti-PD-1 immune checkpoint blockade suppressed tumor growth in vivo, only in the melanoma samples bearing the RAC1^P29S^ mutation. What is important, the tumor is more sensitive to the anti-PD-1 immunotherapy, when the level of CDK9 is low, as its inhibition was correlated with surface expression of PD-L1 proteins. The mechanisms of action of CDK9 in melanoma progression are shown in Figure 5.

### 4.4. The Role of CDK11 in Melanoma

Another important transcriptional kinase is CDK11, existing in over 20 different isoforms, which appear to be an effect of the caspase cleavage or the alternative splicing of the N-terminal coding exons. The three main isoforms, CDK11p110, CDK11p58 and CDK11p46, are able to bind with cyclin L, therefore creating its active form. Similarly to CDK9, this kinase phosphorylates CTD of Pol II and promotes transcription elongation. However, deeper research on biological function of this protein revealed that CDK11 regulates much more significant various processes, like the proper chromosome segregation or pre-mRNA splicing. Therefore, it is also involved in many pathological conditions, including the tau protein phosphorylation in Alzheimer’s disease or replication of HIV-1 virus [109]. 

CDK11 is involved in melanoma pathogenesis, as its expression was significantly increased in the melanoma cells besides the normal melanocytes [40]. The loss of this kinase by siRNAs appeared to arrest the cell cycle of the BRAF and NRAS-mutant cancer cells. Most of the melanoma cells, in which the expression of CDK11 was downregulated, were arrested in the G2/M checkpoint and accumulated in G1 stage, which was probably related to the role of CDK11 in mitosis regulation. What is important, CDK11 had an impact not only on mitotically cycling, but also on non-proliferating, quiescent cells. Furthermore, the loss of CDK11 led to the accumulation of the histone H3 phosphorylation at serine 10, which caused the repression of transcription process and the mitotic arrest. These findings suggest that CDK11 functions in melanocytes as both cell cycle-controlling and transcriptional CDK. To our knowledge, it is the first and only comprehensive article reporting the role of CDK11 in melanoma. Some of the older studies report a heat-shock protein 90 (Hsp90) and its co-chaperone cdc37 stabilize CDK11, therefore regulating its pro-apoptotic function in human melanoma cells [110]. Another research suggest that NOT2 protein induces apoptosis in A375 cells through the same signaling pathway as CDK11 [111]. Moreover, CDK11 was found to promote the Fas-induced apoptosis in melanoma cells through the direct signaling in mitochondria [112]. The deeper investigation in its biological functioning may provide some novel strategies for treating cancer by arresting both cell cycle and the transcription of the cells. 

### 4.5. The Role of CDK12 in Melanoma

**Figure 6:**
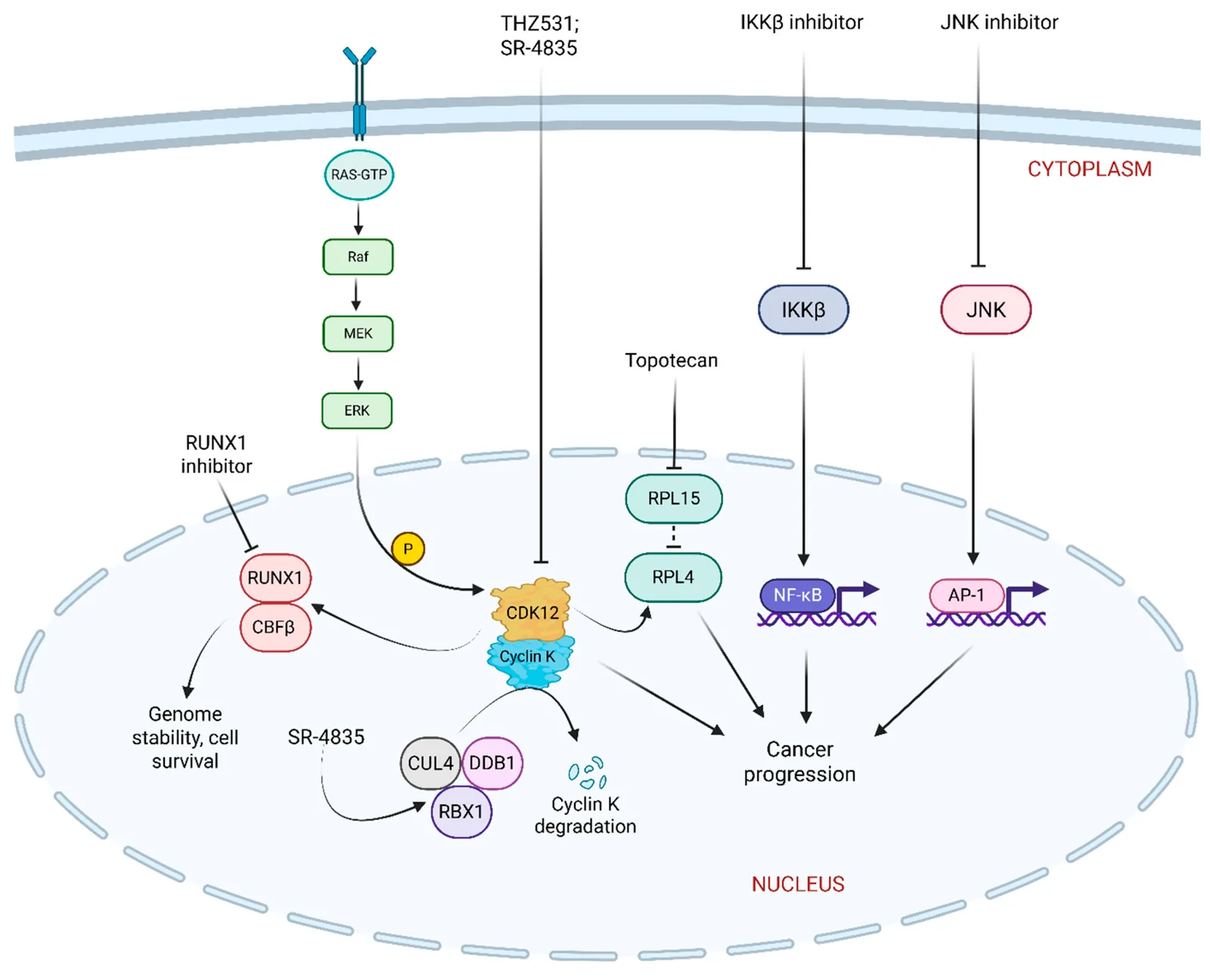
Simplified schematic representation of the signaling pathways associated with CDK12 and Cyclin K in cancer. The diagram illustrates interactions with the RAS/RAF/MEK/ERK, RUNX1/CBFβ, NF-κB, and AP-1 pathways, as well as selected therapeutic inhibitors targeting these networks, highlighting their potential roles in regulating genome stability, cell survival, and cancer progression.

CDK12 plays various oncogenic functions, including metastases activation, deregulation of cellular metabolism and immune response, acquiring drug resistance or sustained proliferation of the cells in many pathological conditions, such as breast, gastric or colorectal cancer [113]. Its activity is regulated by the ERK1/2 proteins of the RAS/MAPK family; therefore, it is highly activated in the melanoma cells (Figure 6) [114]. CDK12 inhibition suppresses the expression of the long genes involved in the DNA repair, concurrently promoting the short-growth-promoting genes expression. As those genes are mostly regulated by the AP-1 and NF-κB transcription factors, the researchers suggested the synergistic inhibition of CDK12 and AP-1/ NF-κB for potential melanoma therapy [114]. Of particular interest was the finding that the SR-4835 inhibitor not only significantly attenuated CTD Pol II phosphorylation in five melanoma lines (Colo829, A375, WM164, WM983A, and WM983B) with BRAF mutations but also promoted cyclin K degradation [51]. This occurs by enhancing the interaction between the CDK12/cyclin K complex and DNA damage binding protein 1 (DDB1), a component of the CUL4-RBX1-DDB1 ubiquitin ligase complex. SR-4835 owes this characteristic “molecular glue” effect, promoting the interactions of these proteins, to its benzimidazole side chain, which is the basis of this drug’s cytotoxicity.

A genome-wide CRISPR-Cas9 screen identified the Runt-related transcription factor 1 (RUNX1) and its cofactor core-binding factor beta subunit (CBFβ) as novel synthetic lethality partners for CDK12 [52]. The RUNX1/CBFβ complex maintained genome stability in A375 melanoma cells in which CDK12 activity had been previously inhibited. Its action, similarly to CDK12, is based on the regulation of genes involved in DNA damage repair; therefore, synergistic inhibition of CDK12 and RUNX1/CBFβ seems to be a promising therapeutic strategy leading to melanoma cell death.

Studies have also shown that CDK12 is able to restore the stability of ribosomal protein L4 (RPL4) in a mouse model of melanoma, thus counteracting the effect induced by topotecan, a topoisomerase I inhibitor, which binds RPL15, inhibiting its interaction with RPL4 [115]. RPL15 and RPL4 are proteins essential for the proper assembly of ribosomal subunits, and disruption of their function results in an enhanced anti-tumor immune response. CDK12 counteracts this effect through an unknown mechanism, suggesting its potential involvement in controlling the immune system’s response to cancer.

### 4.6. The Role of CDK13 in Melanoma

Very few reports also indicate the involvement of CDK13, a kinase with an amino acid sequence 43% identical to CDK12, in the development of melanoma. CDK13 also binds to cyclin K to phosphorylate serine residues on the CTD chain of Pol II [36]. However, the exact role of this kinase still requires much research. Recent reports indicate that, in addition to transcriptional regulation, it is also indirectly involved in the control of apoptosis. CDK13 is involved in the phosphorylation of Nuclear Casein and Cyclin-dependent Kinase Substrate 1 (NUCKS1), resulting in a reduced level of melanoma cell apoptosis [116]. DNA damage induced by oxidative stress led to the reduction in CDK13 through the activation of the ATM/Chk2/Cdc25C axis, which resulted in a decrease in the level of NUCKS1 phosphorylation and increased apoptosis in the A375 and A875 lines [116,117].

Another study by Insco et al. showed that the mutant CDK13 protein is unable to phosphorylate ZC3H14, a factor necessary for the degradation of aberrant mRNA transcripts. The researchers emphasized that mutations in the gene encoding CDK13 contribute to the development of melanoma in zebrafish due to the accumulation of aberrant RNA in the cell nucleus [118].

## 5. The Role of Atypical CDKs in Melanoma

### 5.1. The Role of CDK5 in Melanoma 

In early studies, the role of CDK5 in melanoma development was very unlikely. When the impact of the tyrosine and phenylalanine limitations on B16BL6 melanoma cells proliferation was analyzed, no changes in CDK5 expression were observed [119]. However, immunohistochemistry analysis revealed an increased level of CDK5, along with the classic cyclin-dependent kinases CDK1 and CDK2, in melanoma tissue samples [103]. The melanocytes from normal skin, as well as melanoma in situ, had no sign of the overexpression of the above kinases, whereas melanoma samples in both vertical and metastatic growth phases were characterized with their increased levels. Moreover, the small-molecule CDKI, dinaciclib, targeted at various CDKs, including CDK5, was used in the study. It significantly arrested melanoma cell proliferation and human metastatic melanoma xenografts. 

CDK5 was found to be involved in the phosphorylation of a non-receptor tyrosine Focal Adhesion Kinase (FAK) at serine 732, leading to pathological chromosome alignment during the mitosis [120]. There is a significant function of FAK in the mitotic spindle formation, along with mitosis process of tumor cells. The amount of phosphorylated form of FAK was significantly decreased after the cells were treated with roscovitine, a CDKI targeted mainly at CDK5. There are also few reports on the fact that CDK5 is involved in the regulation of retinoic acid pro-apoptotic effect. Although the research was directly related to the prostate cancer cells, Chen et al. pointed out that retinoic acid has its inhibitory effect on murine and human melanomas, which suggest a potential relation between the CDK5 effect and arresting melanoma cell progression by retinoic acid [121]. However, more research needs to be conducted in order to confirm or reject the existence of this correlation. 

A breakthrough in searching for new melanoma therapeutic targets was when Bisht et al. conducted comprehensive research, using several human melanoma cell lines, including A375 [60,122]. The cells were characterized by the overexpression of both CDK5 and its binding protein p35. Although knockdown of CDK5, mediated by short hairpin RNA (shRNA), did not have any direct impact on melanoma cell growth, it caused changes in cell morphology and significantly arrested the cell motility, invasiveness and reduced colony formation. Analogically, several xenograft analyses proved that CDK5 somehow must be responsible for tumor growth and metastases. The melanoma progression and metastases were also suppressed by the novel CDK5 inhibitor, N-(5-isopropylthiazol-2-yl)-3-phenylpropanamide (PJB). This study not only proved the involvement of CDK5 in metastatic melanoma but also became a starting point for later therapeutic research. According to the analysis by Merk et al., knockdown of CDK5 inhibits the tumor growth of melanoma cells by arresting the non-productive angiogenesis in a mice tumor model [123,124]. 

The alpaca melanocytes, as well as transgenic mice and sheep, were examined in order to discover the mechanisms of production and transportation of melanin [125]. The important melanogenic enzymes-regulating genes—microphthalmia-associated transcription factor (MITF) and paired-box 3 (PAX3)—were found to be regulated by CDK5. The results of CDK5 knockdown analysis demonstrated that this kinase is involved in both normal melanogenesis and pathological processes, including melanoma and other cutaneous diseases. In another study, the interaction between melanoma cells and the HU177 collagen epitope was found to promote the CDK5-dependent signaling [126]. CDK5 regulated the nuclear accumulation of the transcriptional coactivator Yes-associated protein (YAP), which in turn played a key role in melanoma cell migration and metastases, promoted by the HU177 epitope.

CDK5 regulates the motility and metastatic spread of melanoma cells via the phosphorylated form of the transcription factor cAMP response element-binding protein (p-CREB) [61]. A suppression of melanoma cell proliferation and migration was observed after a CDK5 knockdown and correlated with p-CREB downregulation. The research showed that the cell proliferation was mainly related to calcium/calmodulin-dependent kinase 4-p-CREB (CaMK4-p-CREB) pathway, whereas migration seemed to be regulated by p-CREB, integrin beta 1, and integrin beta 5. This study also confirmed the fact that CDK5 is involved in melanogenesis, as its knockdown arrested the tyrosinase activity via regulation of the microphthalmia-associated transcription factor (MITF) and p-CREB, eventually inhibiting melanin production.

Another very remarkable discovery is the fact that CDK5 is involved in tumor immunity, by regulating the programmed cell death-ligand 1 (PD-L1) [127]. PD-L1 is a protein located on the surface of a tumor cell, which can bind with the programmed cell death 1 (PD-1) receptor on T cells, therefore allowing a tumor cell to avoid the immune attack [128]. As CDK5 was found to regulate the PD-1/PD-L1 pathway, Deng et al. decided to project a unique CRISPR-Cas9 system to knock out the CDK5 coding gene in murine melanoma tumor in vivo [62]. They prepared a nano-sized complex of CRISPR-Cas9 plasmids and a biodegradable cationic polymer, poly(β-amino ester) (PBAE), and placed it into melanoma tumors. The knockout of CDK5 coding gene led to downregulation of PD-L1 expression and eventually inhibited tumor growth and lung metastases, creating a new strategy for melanoma treatment, with the use of nanotechnology. 

In another study, CDK5 was knocked out in melanoma patient-derived xenografts and cell lines [63]. The results showed that this kinase does not promote the growth of primary tumors but is involved in the mechanisms of metastatic spread and melanoma cell invasiveness [63,122]. What is important is that this study not only proves the role of CDK5 in metastatic melanoma but also brings us new information on the mechanism in which it works. The deeper, proteome-wide analyses revealed that CDK5 controls the cell invasiveness by the direct phosphorylation of vimentin, a type III intermediate filament protein [63].

**Figure 7:**
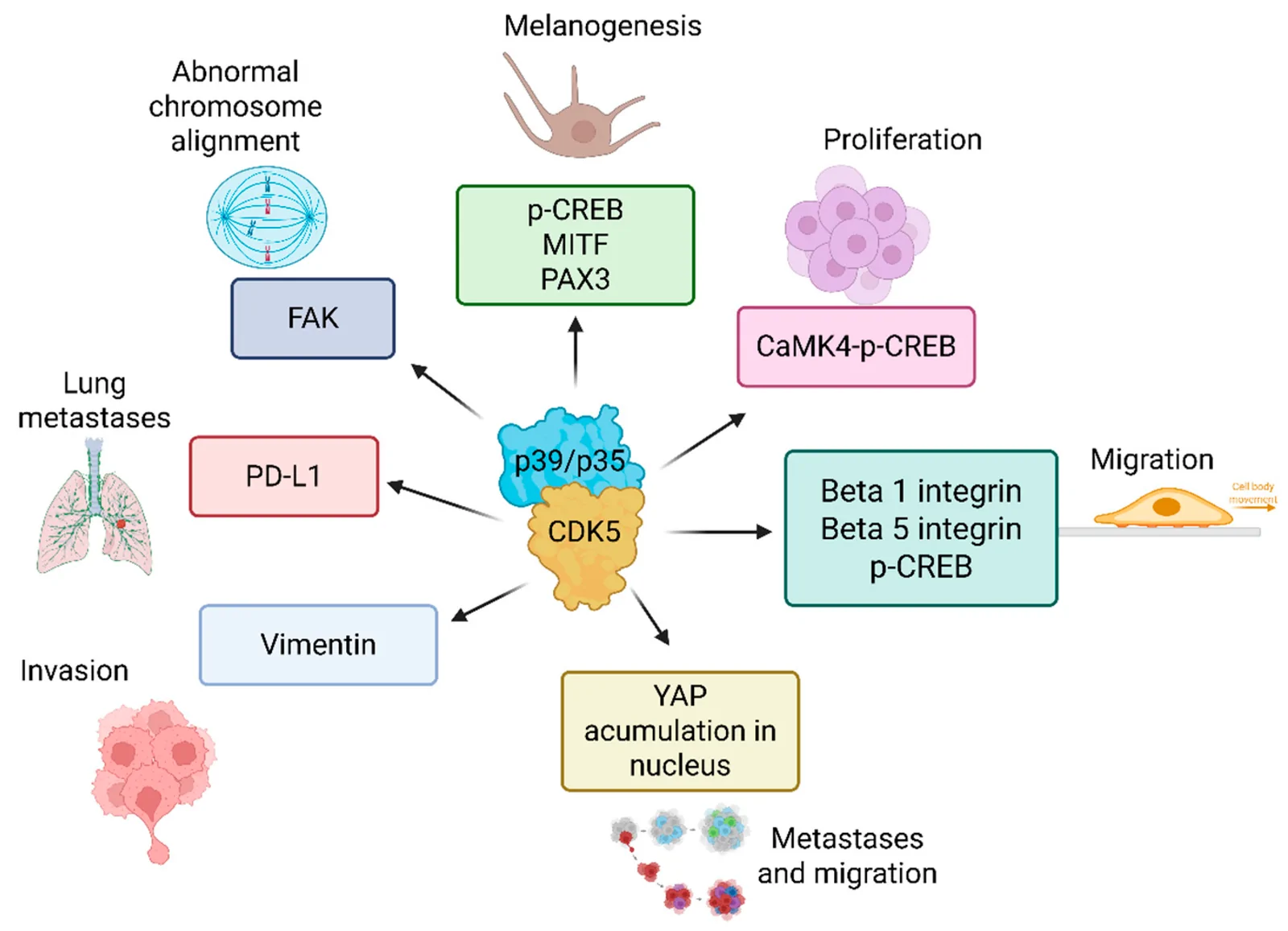
Simplified schematic representation of the signaling pathways associated with CDK5 in melanoma. The figure summarizes the major downstream effectors and biological processes regulated by CDK5, including melanogenesis, proliferation, migration, invasion, metastasis, immune modulation, and chromosome alignment, highlighting its multifaceted role in melanoma progression.

Recent studies provide preliminary evidence for potential involvement of CDK5 in melanoma biology. The heterocyclic steroidal pyrazolo[1,5-α]pyrimidines were found to reduce melanoma cell line proliferation, and the structure–activity relationship analysis revealed their inhibitory effect on protein kinases including CDK5 or its binding proteins, p35 and p25 [129]. In addition, CDK5 expression was reported to be correlated with a long intergenic non-protein coding RNA, LINC00518, associated with a cutaneous melanoma progression [130]. Moreover, in silico study, using a computer-aided drug design platform, identified CDK5 as one of the four most plausible targets for flavonoids against melanoma [131]. However, these findings should be interpreted cautiously, as direct experimental evidence defining the specific contribution of CDK5 to melanoma progression remains limited. Nevertheless, the available data indicate that CDK5 may represent an emerging target in melanoma, particularly in processes beyond canonical cell-cycle regulation. Further studies using genetic and pharmacological approaches are therefore needed to determine whether CDK5 directly regulates melanoma cell proliferation, invasion, metastasis, or other disease-relevant phenotypes and to establish its potential as a therapeutic target. Figure 7 presents all the most important functions that CDK5 plays in melanoma.

### 5.2. The Role CDK16 in Melanoma

Silencing CDK16 expression in melanoma cells was first performed in vivo by Yanagi et al., who used a mouse tumor model and small interfering RNA (siRNA) encapsulated in lipid nanoparticles [64]. This strategy inhibited tumor growth and induced apoptosis in A2058 cancer cells. There were suggestions that silencing CDK16 expression may inhibit cell proliferation by inhibiting processes necessary for cell division, in which this protein is involved.

The identification of CDK16 and NEK9 by Phadke et al. as therapeutic targets for the BRAF inhibitor dabrafenib is a significant finding implicating this kinase in melanoma progression [132]. This study demonstrated that CDK16 is responsible for melanoma progression and poorer prognosis in samples from the Cancer Genome Atlas Program. The mechanism of action of this kinase involves maintaining low levels of the p27 inhibitor and phosphorylation of the Rb protein, leading to excessive proliferation of WM1366 melanoma cells with mutations in the NRAS and KRAS genes. Synergistic inhibition of CDK16 and NEK9 with dabrafenib resulted in reduced tumor cell proliferation, suggesting that CDK16 may be a good therapeutic target for the treatment of BRAF-negative melanoma [132]. Phosphorylation and degradation of the inhibitor protein p27 was later confirmed as an important mechanism in the development of melanoma and other cancers [64,133]. Zhang et al. demonstrated that CDK16 expression in SK-MEL-1, A375, M14, and A2058 melanoma cells is indirectly enhanced by the long non-coding RNA (lncRNA) HCG18, via binding to the HCG18 target sequence miR-324-5p [65]. Similar to previous studies, high levels of CDK16 promoted melanoma cell proliferation, migration, and invasiveness. In the latest computer analysis of BRAF phosphorylation sites, CDK16 was identified as one of the main proteins responsible for the phosphorylation of this kinase [134].

## 6. Conclusions and Future Prospects

Melanoma treatment poses a significant challenge for modern medicine. Although the development of BRAF-targeted therapies and anti-PD-1 antibodies has substantially improved outcomes for many patients, durable responses are not achieved in all cases, and effective therapeutic options remain limited for melanomas lacking actionable mutations or those that have acquired drug resistance. Because CDKs control fundamental processes essential for proper cellular function, they have long been analyzed for potential therapeutic targets in melanoma. Although classic CDKs primarily regulate the cell cycle, we can also distinguish CDKs that regulate DNA transcription and atypical CDKs.

The atypical CDK5 kinase, known for its multifunctional role in controlling the nervous system, immediately attracts considerable attention. It is noteworthy that CDK5 rarely significantly influences the growth of cancer cells themselves, but it significantly contributes to the migration and invasiveness of melanoma cells, as well as the occurrence of metastasis. Numerous studies have confirmed the involvement of CDK5 in melanoma cell migration, which was associated with the activity of other proteins such as p-CREB and MITF. CDK5 has also been shown to inhibit cancer cell apoptosis via the PD-1/PD-L1 pathway and promote their invasiveness through vimentin phosphorylation. Another atypical kinase, CDK16, promotes excessive melanoma cell proliferation through the reduction in p27 levels and the phosphorylation of Rb protein. 

Transcriptional CDKs appear to be equally important factors. The expression of the CAK complex protein, CDK7, is enhanced in melanocytes by the proto-oncogenes MITF and c-MYC. It is also known to influence the invasiveness of uveal melanoma cells and the formation of liver metastases, which is related to the activity of RhoA GTPase. CDK7 activity is closely linked to the CDK9 activity, which is not coincidental, as only after phosphorylation of the T-loop by CAK is CDK9 able to activate transcriptional elongation. However, despite evidence of its involvement in tumor development, inhibition of this protein should not be used as a monotherapy, as it may underlie the emergence of drug resistance and cancer cell survival through GATA 6 transcription program. Therefore, future use of CDK7 as a therapeutic target in melanoma treatment would necessarily involve the use of synergistic therapies.

Studies on CDK9 have shown that this protein controls the expression level of the MDM4 proto-oncogene, a negative regulator of the p53 tumor suppressor. Moreover, inhibition of CDK9 stops the growth of melanoma cells with the BRAFV600E mutation, which are resistant to BRAF/MEK inhibitors. Also, in cutaneous and uveal melanoma without mutations in the BRAF, NRAS and NF1 genes, CDK9 inhibition inhibits the cell cycle in the G1/S phase, indirectly through E2F family proteins, and kills cancer cells. This protein also influences cell apoptosis and tumor microenvironment. In turn, CDK11 inhibition stops both transcription due to histone H3 phosphorylation and the cell cycle in dividing and dormant cells. 

The role of one of the Mediator components, CDK8, in melanoma was once associated only with its inverse correlation with mH2A and cell cycle arrest in the G2/M phase. However, we now know that CDK8 may also contribute to melanoma progression by phosphorylating STAT1 and increasing the cytotoxicity of NK cells. Research on the role of this kinase is relatively old, and there are no current reports in the literature on its use as a therapeutic target. The probable reason for this is the ambiguous function of Mediator―both inhibiting and activating transcription—which makes the use of CDK8 in therapy difficult. Research on this protein should be refreshed and updated to gain a more accurate understanding of its functions. CDK12 contributes to the proliferation of melanoma cells by controlling the expression of genes involved in DNA repair, and its inhibition with the inhibitor SR-4835 further degrades cyclin K by enhancing the interaction with DDB1, a component of ubiquitin ligase. 

Despite the growing evidence supporting the potential involvement of transcriptional and atypical CDKs in melanoma, the available data remain limited, and many aspects of their biological functions and therapeutic relevance are still poorly understood. The limitations result mainly from the fact that research on such therapies is currently mainly of a preclinical form. Understanding the signaling pathways in which individual CDKs are involved, as well as their functions and the effectiveness of CDKs in vitro, is only the first step towards creating new therapeutic methods. It is necessary to find appropriate synergies that will guarantee the full effectiveness and safety of CDKI, as well as clinical trials that will reveal the actual impact of the therapy on the patient’s body. Another problem is the relatively small number of selective CDKIs targeting atypical CDKs, compared to classical CDKs, which are targeted by numerous inhibitors regularly used in clinical practice. Besides that, CDK7 inhibitors have entered clinical trials, with gastrointestinal and hematological toxicities representing important dose-limiting considerations, although most adverse events were manageable [135]. Similarly, clinical studies of the CDK9 inhibitors have demonstrated pharmacodynamic activity but also frequent neutropenia and other laboratory abnormalities, highlighting the importance of maintaining an adequate therapeutic window [136].

However, it should be remembered that initial analyses of the functions of individual proteins in normal and pathological conditions are an essential element for further considerations and analyses leading to the implementation of new therapeutic methods in the fight against cancer progression. Therefore, the role of such important enzymes as atypical and transcriptional CDKs should not be ignored, as in the coming years they may constitute new targets for therapy that can overcome current barriers in the treatment of melanoma. 

## Figures and Tables

**Table 1:** Comparison of the characteristics and functions of transcriptional CDKs.

CDK	Cyclins (Complex)	Transcription Stage	Function in Transcription	CTD Phosphorylation Sites	References
CDK7	Cyclin H, MAT1 (CAK, TFIIH)	Initiation	Activating: release of Pol II from the promoter; T-loop phosphorylation–CDK activation	Serine 5; Serine 7	[5,8]
CDK8/CDK19	Cyclin C, MED12/13 (CDK8 module, Mediator)	Initiation	Inhibitory: blockade of Pol II binding to the pre-initiation complex; Activating: activation of BRD4 and P-TEFb	Serine 2; Serine 5	[5,12]
CDK9	Cyclin T (P-TEFb)	Pause before elongation	Activating: CTD phosphorylation, inhibition of DSIF and NELF	Serine 2	[8]
CDK10	Cyclin M	No data	Inhibitory: negative regulation of ETS2-dependent transcription	No data	[5]
CDK11	Cyclin L	Elongation; splicing	Activating: control of histone protein expression; regulation of splicing, mitosis and apoptosis; interaction with TFIIF and TFIIS	Serine 2	[12]
CDK12/CDK13	Cyclin K	Elongation; termination; splicing	Activating: increasing Pol II processivity and speed; blocking intronic alternative polyadenylation sites	Serine 2	[13]

**Table 2:** Preclinical studies from the last 10 years targeting transcriptional CDKs in melanoma progression.

CDK Activity Modulator	CDK	Main Effect After Drug Application	Source
THZ1	CDK7	Reduction in MITF and SOX10 expression through disruption of super-enhancers	[42]
SNS-032	CDK7/CDK9	Inhibition of YAP, KLF4, MMP9, c-MYC and RhoA; induction of apoptosis; reduction in invasiveness and liver metastases in uveal melanoma lines/models	[43]
THZ1	CDK7	Inhibition of MITF; promotion of GATA6-regulated tolerance to BRAF/MEK inhibitors	[44]
CDKI-73	CDK9	Synergy with BRAF/MEK and HDAC inhibitors; induction of apoptosis; inhibition of melanoma model growth	[45]
Dinaciclib, flavopiridol, roscovitine, AT-7519, SNS-032, DRB, atuveciclib	CDK9	Synergy of atuveciclib with an MDM2 inhibitor; reduction in MDM4 level; activation of p53; inhibition of cancer cell proliferation	[46]
TS-032, NVP-2	CDK9	Inhibition of E2F factors and cell-cycle arrest in BRAF/NRAS/NF1 WT cutaneous and uveal melanoma cells	[47]
CDKI-73	CDK9	Inhibition of anti-apoptotic proteins BCL2, BCLXL and MCL1 expression	[48]
LS-007	CDK9	Polarization of macrophages toward an anti-tumor phenotype in the tumor microenvironment; apoptosis	[49]
Dinaciclib	CDK9	Synergy with anti-PD-1; inhibition of proliferation of melanoma cells with the RAC1P29S mutation	[50]
siRNA	CDK11	Accumulation of the unphosphorylated form of histone H3 at Ser10; transcriptional repression and G2/M cell-cycle arrest	[40]
THZ531, SR-4835	CDK12	Promotion of expression of short growth-promoting genes; synergy with AP-1 and NF-κB inhibitors; inhibition of BRAF-mutant melanoma	[37]
SR-4835	CDK12	Cyclin K degradation through integration with DDB1; inhibition of Pol II CTD phosphorylation	[51]
THZ531, SR-4835	CDK12	Synergy with RUNX1/CBFβ inhibition; impaired DNA repair; melanoma cell death	[52]

**Table 3:** Comparison of the characteristics and functions of atypical CDKs.

CDK	Subgroup	Partner Proteins	Expression Area/Regulated Processes	Main Pathological Conditions	References
CDK5	PSSALRE	p35, p39, cyclin B1	Nervous system: neuronal migration, neurogenesis, synaptic plasticity, cytoskeleton organization	Neurodegenerative diseases: Alzheimer’s disease/Parkinson’s disease; cancers: breast, lung and gastric cancer	[53]
CDK14	PFTAIRE	Cyclin Y, cyclin D3	Nervous system: stimulation of the Wnt pathway	Cancers: hepatocellular carcinoma, pancreatic cancer	[5]
CDK15	PFTAIRE	No data	Gonads: role unknown	Cancers: colorectal cancer	[5]
CDK16	PCTAIRE	Cyclin Y, CCNYL1	Various tissues: spermatogenesis, apoptosis, autophagy, cytoskeleton organization, myocyte maturation	Cancers: lung adenocarcinoma, lung squamous cell carcinoma; neuropsychological disorders	[54,55]
CDK17	PCTAIRE	No data	Role unknown	Alzheimer’s disease; cancers	[54]
CDK18	PCTAIRE	Cyclin A2, cyclin E2	Vesicular transport, neurogenesis	Cancers: gastric cancer	[54]
CDK20	PNQALRE	Cyclin H	CAK activity for CDK2, involvement in the Wnt pathway	Cancers: ovarian, brain, colorectal, gastric, liver and lung cancer	[56]

**Table 4:** Studies from the last 10 years on modulation of atypical CDK activity in melanoma cell lines or models.

CDK Activity Modulator	CDK	Main Effect of Activity Modulation	Source
siRNA	CDK5	Reduction in CaMK4-p-CREB pathway activity; inhibition of proliferation and migration	[61]
CRISPR-Cas9	CDK5	Reduction in PD-L1 expression; abolition of liver metastases	[62]
CDK5-knockout	CDK5	Inhibition of cell proliferation and tumor growth in mice	[63]
Lipid nanoparticle-encapsulated siRNA	CDK16	Inhibition of melanoma cell growth	[64]
Dabrafenib	CDK16	Synergy with a NEK9 inhibitor; increased p27 level	[64]
si-HCG18	CDK16	Reduction in HCG18 lncRNA and CDK16 levels; melanoma cell apoptosis	[65]

## Data Availability

No new data were created or analyzed in this study. Data sharing is not applicable to this article.

## Abbreviations

AP-1	Activator protein 1
BETs	Bromodomain and extra-terminal domain proteins
BRAF	B-Raf serine/threonine kinase
BRD4	Bromodomain-containing protein 4
CAK	CDK-activating kinase
CaMK4	Calcium/calmodulin-dependent protein kinase type IV
CBFβ	Core-binding factor subunit beta
CDK	Cyclin-dependent kinase
CDKI	Cyclin-dependent kinase inhibitor
CTD	C-terminal domain of RNA polymerase II
DDB1	DNA damage-binding protein 1
DSIF	DRB sensitivity-inducing factor
ERK	Extracellular signal-regulated kinase
FAK	Focal adhesion kinase
FUBP2	Far upstream element-binding protein 2
GATA6	GATA-binding factor 6
GSEA	Gene set enrichment analysis
GTP	Guanosine triphosphate
HDAC	Histone deacetylase
Hsp90	Heat shock protein 90
KLF4	Krüppel-like factor 4
lncRNA	Long non-coding RNA
MAPK	Mitogen-activated protein kinase
MAT1	Ménage-à-trois 1 (CDK-activating kinase assembly factor 1)
MDM4	Mouse double minute 4
mH2A	Macro-H2A histone
MITF	Microphthalmia-associated transcription factor
MMP9	Matrix metalloproteinase-9
NELF	Negative elongation factor
NF-κB	Nuclear factor kappa-light-chain-enhancer of activated B cells
NUCKS1	Nuclear casein kinase and cyclin-dependent kinase substrate 1
PARP1	Poly (ADP-ribose) polymerase 1
PAX3	Paired box 3
p-CREB	Phosphorylated cAMP response element-binding protein
PD-1	Programmed cell death protein 1
PD-L1	Programmed death-ligand 1
PI3K	Phosphoinositide 3-kinase
Pol II	RNA polymerase II
PRAME	Preferentially expressed antigen in melanoma
P-TEFb	Positive transcription elongation factor b
PTEN	Phosphatase and tensin homolog
Rb	Retinoblastoma protein
RPL4	Ribosomal protein L4
RUNX1	Runt-related transcription factor 1
shRNA	Short hairpin RNA
siRNA	Small interfering RNA
snRNP	Small nuclear ribonucleoprotein
STAT1	Signal transducer and activator of transcription 1
TGF-β	Transforming growth factor beta
YAP	Yes-associated protein
